# Neutralization of the SARS-CoV-2 Delta variant after heterologous and homologous BNT162b2 or ChAdOx1 nCoV-19 vaccination

**DOI:** 10.1038/s41423-021-00755-z

**Published:** 2021-08-23

**Authors:** Swantje I. Hammerschmidt, Berislav Bosnjak, Günter Bernhardt, Michaela Friedrichsen, Inga Ravens, Alexandra Dopfer-Jablonka, Markus Hoffmann, Stefan Pöhlmann, Georg M. N. Behrens, Reinhold Förster

**Affiliations:** 1 Institute of Immunology, Hannover Medical School, Hannover, Germany; 2 Department for Rheumatology and Immunology, Hannover Medical School, Hannover, Germany; 3 Infection Biology Unit, German Primate Center—Leibniz Institute for Primate Research, Göttingen, Germany; 4 Faculty of Biology and Psychology, Georg-August-University Göttingen, Göttingen, Germany; 5 German Center for Infection Research (DZIF), Partner Site Hannover-Braunschweig, Hannover, Germany; 6 German Center for Lung Research (DZL), Hannover, Germany; 7 Cluster of Excellence RESIST (EXC 2155), Hannover Medical School, Hannover, Germany

**Keywords:** Antibodies, Vaccines

Since the beginning of the COVID-19 pandemic, divergent variants of concern (VoCs) of SARS-CoV-2 have evolved and become the most prevalent SARS-CoV-2 variants in distinct locations at different times. Currently, the Delta variant (B.1.617.2) dominates infection events in large parts of the world. Immunization campaigns, however, still use SARS-CoV-2 vaccines based on the spike (S) protein of the original Wuhan virus.

The S protein of the Delta variant of SARS-CoV-2 harbors mutations that support replication and transmission but also weaken the binding of neutralizing antibodies. The Delta variant has been reported to evade control by antibodies induced upon infection and, arguably more relevant, after BNT162b2 (BNT) vaccination [1, 2]. Likewise, the ChAdOx1 nCoV-19 (ChAd) vaccine appeared less effective than the BNT vaccine in preventing SARS-CoV-2 infection with the Delta variant [2, 3].

In addition to homologous prime-boost protocols, millions of ChAd-primed vaccinees received heterologous boost immunization with mRNA-based SARS-CoV-2 vaccines, as vaccination with ChAd was halted due to an increased risk of thrombotic events.

The results from randomized [4, 5] and observational studies [6–8] demonstrated that heterologous prime-boost protocols also induce robust humoral and cellular responses accompanied by acceptable reactogenicity. These reports prompted others to suggest that mixing vaccines might be a suitable strategy to combat emerging SARS-CoV-2 variants [9]. However, information is limited regarding the neutralization capacities against the Delta variant of various immunization regimens.

We assessed plasma from 85 individuals at a mean of 68 days (range 45–91 days) after ChAd priming and a mean of 17 days (range 13–23 days) after either homologous ChAd (*n* = 31, 20 females) or heterologous BNT (*n* = 54, 40 females) prime-boost protocols [6] for their capacity to neutralize the Delta variant by applying surrogate virus neutralization tests (sVNTs). For comparison, we also tested plasma from 30 individuals (21 females) at a mean of 21 days (range 18–27 days) after BNT priming and at a mean of 30 days (range 15–65 days) after homologous BNT prime-boost protocols.

While homologous ChAd boosting only slightly increased neutralization of the Delta variant, heterologous ChAd/BNT vaccination led to a ninefold increase in neutralizing titers (Fig. 1a), resulting in detectable neutralizing sVNT titers in all individuals receiving this vaccination schedule. Similarly, homologous BNT prime-boost protocols also led to a ninefold increase but resulted in overall higher titers of neutralizing antibodies than heterologous immunization (Fig. 1b). As reported before for the Alpha, Beta, and Gamma VoCs [6], the results obtained with the Delta receptor binding domain-based sVNT were also closely correlated with data obtained with a vesicular stomatitis virus-based pseudotyped virus neutralization assay [10], which is based on particles harboring the spike protein of the Delta variant (Fig. 1c). Interestingly, although heterologous BNT boost after ChAd prime consistently resulted in higher neutralizing titers against the Alpha, Beta, and Gamma variants compared to homologous BNT vaccination [6], homologous BNT prime-boost appears to more efficiently induce neutralizing antibodies against the Delta variant.

**Fig. 1:**
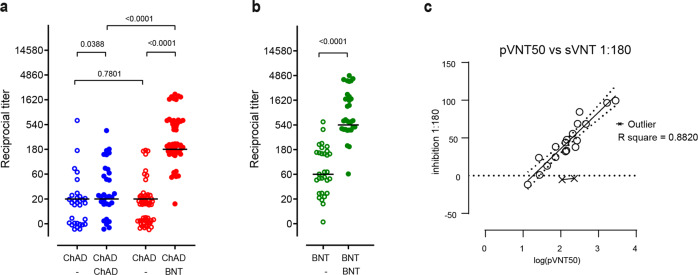
Stronger humoral immune responses against the Delta SARS-CoV-2 variant following heterologous ChAdOx1 nCoV-19 (ChAd)/BNT162b2 (BNT) than homologous ChAd/ChAd vaccination. **a** Reciprocal titers of neutralizing antibodies against the Delta SARS-CoV-2-S variant measured using a surrogate virus neutralization test (sVNT). Data are from *n* = 31 biologically independent samples from the ChAd/ChAd group and *n* = 54 biologically independent samples from the ChAd/BNT group. **b** Reciprocal titers of neutralizing antibodies against the Delta SARS-CoV-2 variant measured using a surrogate virus neutralization test (sVNT). Data are from *n* = 30 biologically independent samples from BNT/BNT. **c** Efficacies of antibody neutralization against the SARS-CoV-2 Delta variant measured by surrogate virus neutralization tests (sVNT) show a strong positive correlation with those of pseudotyped virus neutralization tests (pVNT). Correlation (solid line) and 95% confidence intervals (dotted lines) between sVNT_1:180_ and antibody titers resulting in a 50% reduction in luciferase activity in pVNT, indicated as pVNT_50_. Open circles, values from individual donors, and outliers are marked with X and were defined as values with absolute residual value >2 SD of all residual values in each group of samples. Correlation was calculated using simple linear regression. **a**, **b** Chi-square test for trend; dots represent individual vaccinees, lines represent group median. Open symbols: preboost sera, filled symbols: postboost sera. For better visualization of identical titer values, data were randomly and proportionally adjusted closely around the precise titer result

The overall robust inhibition of the Delta variant further supports heterologous boosting with BNT of vaccinees initially primed with ChAd. However, in contrast to Alpha, Beta, and Gamma variants, homologous BNT prime-boost vaccination might be even more efficient in neutralizing the Delta variant.

Our data emphasize a high level of complexity in antibody responses that is affected not only by the vaccine or the vaccinee’s immune system but also by the VoC to be combatted. Moreover, our data indicate the urgent need for detailed studies to evaluate which vaccine schedules are best suited to combat COVID-19, once humoral immunity starts diminishing and the risk for infection and disease rises again in the vaccinated population.

## Supplementary information

Supplementary Material and Methods
