# Supplementary material for: Neutralization of the SARS-CoV-2 Delta variant after heterologous and homologous BNT162b2 or ChAdOx1 nCoV-19 vaccination
Source: Cell Mol Immunol. 2021 Aug 23;18(10):2455–6. doi: 10.1038/s41423-021-00755-z (PMC8381713; doi:10.1038/s41423-021-00755-z)
Supplement: Supplementary file 1 — Supplementary Material and Methods [file 41423_2021_755_MOESM1_ESM.pdf]

Supplementary Information

Neutralization of the SARS-CoV-2 Delta variant after heterologous and homologous BNT162b2 or ChAdOx1 nCoV-19 vaccination

Swantje I. Hammerschmidt[1], Berislav Bosnjak[1], Günter Bernhardt[1], Michaela Friedrichsen[1], Inga Ravens[1], Alexandra Dopfer-Jablonka[2], Markus Hoffmann[3], Stefan Pöhlmann[3,4], Georg M.N. Behrens[2,5,#], Reinhold Förster[1,5,6,7#]

[1] Institute of Immunology, Hannover Medical School, 30625 Hannover, Germany
[2] Department for Rheumatology and Immunology, Hannover Medical School, 30625 Hannover, Germany
[3] Infection Biology Unit, German Primate Center – Leibniz Institute for Primate Research, 37077 Göttingen, Germany
[4] Faculty of Biology and Psychology, Georg-August-University Göttingen, 37073 Göttingen, Germany
[5] German Center for Infection Research (DZIF), Partner Site Hannover-Braunschweig, 30625 Hannover, Germany
[6] German Center for Lung Research (DZL), Hannover, 30625 Hannover, Germany
[7] Cluster of Excellence RESIST (EXC 2155), Hannover Medical School, 30625 Hannover, Germany

[#] equal contribution

Corresponding author:

Reinhold Förster, Institute for Immunology, Hannover Medical School, Carl-Neuberg-Straße 1, D - 30625 Hannover, Germany, Tel: +49 511 532 9721, Fax: +49 511 532 9722, Email: foerster.reinhold@mh-hannover.de

Conflict of Interest:

All authors declare no conflict of interest

Material and Methods

Participants.

The participants of this study have been described in detail before [1]. In brief, participants were from the COVID-19 Contact (CoCo) Study (German Clinical Trial Registry, DRKS00021152), which started in March 2020 and is an ongoing, prospective observational study monitoring anti-SARS-CoV-2 IgG immunoglobulin and immune responses in n=1,493 health care professionals (HCP) at Hannover Medical School and individuals with potential contact to SARS-CoV-2 [2, 3]. An amendment from Dec 2020 allowed the analysis of immune responses after COVID-19 vaccination. The first doses of the BNT vaccine were applied Jan 6th, and of the ChAd vaccine Feb 16th, 2021 onwards. All 30 BNT primed individuals received a BNT booster in general 21 days after priming. Individuals primed with ChAd could choose between ChAd and BNT for booster vaccination that was given 2-3 months after prime. Of the 85 individuals primed with ChAd, 31 chose homologous and 54 chose heterologous immunization during the period of investigation. Peripheral blood samples were drawn by venipuncture after written informed consent was obtained. Participants were 25% male and 75% female with a mean age of 38 years (range 19-64 years) and were representative for all vaccinees of the CoCo Study (72% females, 28% male; mean age 40 years, range 19-67 years). Plasma was separated from EDTA or lithium heparin blood (S-Monovette, Sarstedt) and stored at -80 °C until use.

Pseudotyped virus neutralization assay (pVNT).

pVNTs were performed as described recently [1, 4]. In brief, rhabdoviral pseudotyped particles were produced in 293T cells transfected to express the spike protein of the Delta variant. These cells were inoculated with a replication-deficient VSV vector that encodes for enhanced green fluorescent protein and firefly luciferase (FLuc) instead of VSV-G protein. Serially diluted heat-inactivated (56 °C, 30 min)

plasma samples and equal volumes of purified pseudotyped particles were incubated for 30 min at 37 °C. These samples together with non-plasma incubated pseudotyped particles were used for the transduction experiments that were done using Vero cells in 96-well plates. Transduction efficacy was analyzed 16-18 hr later by determining FLuc activity in lysed cells using a commercial substrate (Beetle-Juice, PJK).

Surrogate virus neutralization assay (sVNT) for SARS-CoV-2 variants.

To determine neutralizing antibodies against the B.1.617.2-Spike (Delta) variant of concern of SARS-CoV-2-S in plasma, we adapted our recently established surrogate virus neutralization test (sVNT) [1, 5] for the Delta variant. In brief, in this enzyme-linked immunosorbent assay (ELISA)-based test, the soluble receptor for SARS-CoV-2, ACE2, is bound to 96-well-plates to which a receptor binding domain (RBD) of the Delta Spike-protein with a C-terminal His-Tag binds once added to the plates. Binding is visualized by an anti-tag peroxidase-labelled antibody and colorimetric quantification. Pre-incubation of the Spike-protein with plasma of vaccinees prevents subsequent binding to ACE2 to various degrees, depending on the amount of neutralizing antibodies present. The sVNT has been performed exactly as described in detail before [1] using 1.5 ng recombinant SARS-CoV-2 Spike RBD of the Delta variant (SinoBiological).

Statistics.

For sVNT statistical analysis was done using GraphPad Prism 8.4 (GraphPad Software, USA) applying the Chi-square test for trend. Differences were considered significant if $p < 0.05$. Correlation between sVNT and pVNT values was calculated using simple linear regression analysis.

Ethics committee approval.

The CoCo Study and the analysis conducted for this article were approved by the Internal Review Board of Hannover Medical School (institutional review board no. 8973_BO-K_2020, amendment Dec 2020).

References

1. Barros-Martins J, *et al.* Immune responses against SARS-CoV-2 variants after heterologous and homologous ChAdOx1 nCoV-19/BNT162b2 vaccination. *Nature Medicine* 2021; https://www.nature.com/articles/s41591-021-01449-9

2. Behrens GMN, *et al.* Perceived versus proven SARS-CoV-2-specific immune responses in health-care professionals. *Infection* 2020; **48**: 631-634.

3. Jablonka A, *et al.* Protocol for longitudinal assessment of SARS-CoV-2-specific immune responses in healthcare professionals in Hannover, Germany: the prospective, longitudinal, observational COVID-19 Contact (CoCo) study. *medRxiv* 2020; https://www.medrxiv.org/content/10.1101/2020.12.02.20242479v1.full

4. Hoffmann M, *et al.* SARS-CoV-2 variants B.1.351 and P.1 escape from neutralizing antibodies. *Cell* 2021; **184**: 2384-2393.e2312.

5. Bosnjak B, *et al.* Low serum neutralizing anti-SARS-CoV-2 S antibody levels in mildly affected COVID-19 convalescent patients revealed by two different detection methods. *Cell Mol Immunol* 2021; **18**: 936-944.
